# Changes in the morphology and protein expression of germ cells and Sertoli cells in plateau pikas testes during non-breeding season

**DOI:** 10.1038/srep22697

**Published:** 2016-03-04

**Authors:** Ming Liu, Guangming Cao, Yanming Zhang, Jiapeng Qu, Wei Li, Xinrong Wan, Yu-xia Li, Zhibin Zhang, Yan-ling Wang, Fei Gao

**Affiliations:** 1 State Key Laboratory of Stem Cell and Reproductive Biology, Institute of Zoology, Chinese Academy of Sciences, Beijing 100101, P.R. China; 2 State Key Laboratory of Integrated Management of Pest Insects and Rodents, Institute of Zoology, Chinese Academy of Sciences, Beijing 100101, P.R. China; 3 Key Laboratory of Qinghai-Tibetan Plateau Biological Adaptation and Evolution, Northwest Institute of Plateau Biology, Chinese Academy of Sciences, Xining, 810008, Qinghai Province, P.R. China; 4 University of Chinese Academy of Sciences, Beijing 100049, P.R. China

## Abstract

Plateau pikas are seasonally breeding small herbivores that inhabit the meadow ecosystem of the Qinghai-Tibetan Plateau. Testis regression in plateau pikas begins in early June, and the male pikas are completely infertile, with a dramatically reduced testis size, in late July. In this study, a decreased germ cell number in the testes was first noted in early June. By late June, only Sertoli cells and a small number of spermatogonia remained. Interestingly, large gonocyte-like germ cells were observed in early July. In late July, the number of gonocyte-like cells per tubule increased significantly, and most of the Sertoli cell nuclei moved to and clustered in the center of the seminiferous tubules. The gonocyte-like germ cells and Sertoli cells began to express AP-2γ and anti-Mullerian hormone (AMH) proteins, which were detected in the germ cells and Sertoli cells of juvenile pikas but not in adult testes. Simultaneously, LC3 puncta dramatically increased in the seminiferous tubules of the pikas’ testes during the non-breeding season. Our study found that spermatogonia and Sertoli cells in non-breeding adult pikas morphologically resembled those in juvenile pikas and expressed specific markers, indicating that de-differentiation-like transitions may occur during this process.

To avoid high death rates of newborn offspring due to adverse environments, most mammals exhibit seasonal reproduction. The daily photoperiod is considered to be the most important environmental factor controlling seasonal breeding^1^,^2^. For example, a short- or long-day photoperiod can induce testis regression or growth in golden hamsters^3^. The downstream molecule triggered by the daily photoperiod is melatonin^4^,^5^. The administration of melatonin can mimic the effects of short-day photoperiods in some mammals^6^,^7^. It has been reported that melatonin is able to influence androgen production and normal spermatogenesis via cross-talk with the hypothalamus-pituitary-gonad (H-P-G) axis^8^, leading to testicular regression during the non-breeding season.

During the non-breeding season, testis regression is observed, during which only spermatogonia, Sertoli cells and some spermatocytes remain in the seminiferous tubules^9^. This process is most likely a consequence of the suppression of the H-P-G axis and a lack of androgen production. Most seasonally breeding mammals, e.g., grey squirrels and rock hyraxes^10^,^11^, follow this pattern. In roe deer and golden hamsters, the regression of seminiferous tubules is more severe and only Sertoli cells and spermatogonia remain^12^,^13^. These results suggest that, in addition to the suppression of the H-P-G axis, the regression of testes may also be influenced by species-specific factors.

Pikas are seasonally breeding small mammals belonging to the order Lagomorpha, family Ochotonidae. In China, most pikas live in or around the Tibetan Plateau, Inner Mongolia and the Xinjiang Autonomous Region. The most common pika in China is the plateau pika *(Ochotona curzoniae)*. It is found in widely scattered locations within and around the Tibetan Plateau^14^. The breeding season of plateau pikas is between April and July^15^, and no newborn pikas are observed after July. In fact, the regression of testes in these pikas occurs in early June, and the testes of most adult male pikas are severely regressed in July. Most juvenile pikas are born in May, and their body sizes reach nearly that of an adult pika by the end of the breeding season^15^. However, juvenile male pikas will not mate during the first year of life because these juveniles’ gonads are still immature. Male pikas start to breed in the following year. A large proportion of adult pikas die at the end of the first breeding season. Approximately 15–40% of adult male pikas survive to the next breeding season^15^. Mature testes are observed in adult male pikas in early April and start to regress in mid-June. The males are completely infertile by late June^16^. The histological changes in pika testes during the transition from the breeding season to the non-breeding season have never been studied.

In this study, we found not only germ cell loss but also changed morphology and protein markers in both germ cells and Sertoli cells in pikas. Large gonocyte-like germ cells were observed, and these cells in the pika testes expressed the gonocyte-specific gene AP-2γ during the non-breeding season. The nuclei of the Sertoli cells moved to the center of the seminiferous tubules and expressed anti-Mullerian hormone (AMH). These results suggest that both germ cells and Sertoli cells most likely undergo a de-differentiation-like (DDL) transition in the non-breeding season in pikas.

## Results

### The size of the testes of plateau pikas decreased dramatically during the non-breeding season

**Figure 1:**
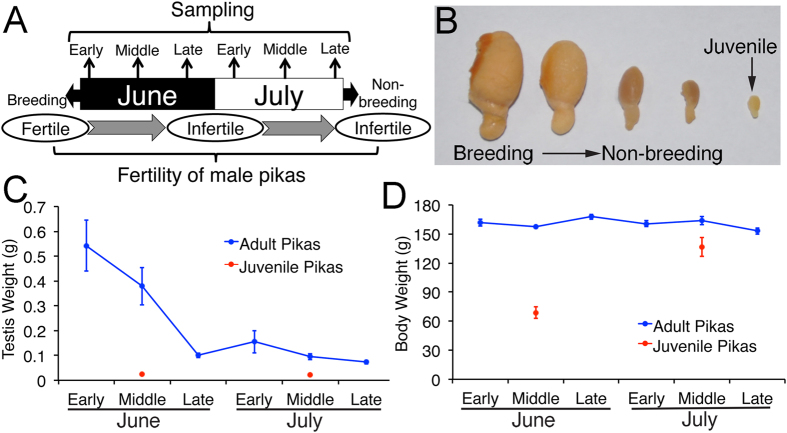
The body and testis weights of plateau pikas during the transition from the breeding to the non-breeding season. (**A**) Schematic representation of the fertility state of plateau pikas and the time points when collections were performed during the transition from the breeding to the non-breeding season. (**B**) The testes of the plateau pikas dramatically decreased in size during the transition from the breeding to the non-breeding season. The weight was higher than that of juvenile pikas. (**C**) The weight of adult pikas’ testes decreased significantly from early June to late June, whereas the weight of juvenile pikas’ testes did not change significantly. (**D**) The body weight of adult plateau pikas was stable from early June to late July, whereas the body weight of juvenile pikas dramatically increased during this period. The bars in panel (**C,D**) represent ± SEM. For adult pikas, n = 10 at each time point; for juvenile pikas, n = 5 in mid-June and mid-July.

Plateau pikas are seasonally breeding small herbivores. The seminiferous cycle of the testes of plateau pikas during the breeding season was examined in this study (Supplementary Figure 1). Based on the combinations of cell types in the seminiferous tubules, the seminiferous cycle was essentially divided into 7 stages. In adult pikas, the size of pika testes was dramatically reduced during the non-breeding season. The average weight of an adult pika testis was approximately 0.5 g in early June but only approximately 0.1 g in late June and July (Fig. 1B,C). In contrast, the body weight of adult pikas remained unchanged from early June to late July (Fig. 1D). The body weight of juvenile pikas increased dramatically from early June to late July, whereas the size of the testis did not change significantly during this period (Fig. 1C,D).

### Histological changes in the seminiferous tubules of plateau pikas from the breeding season to the non-breeding season

**Figure 2:**
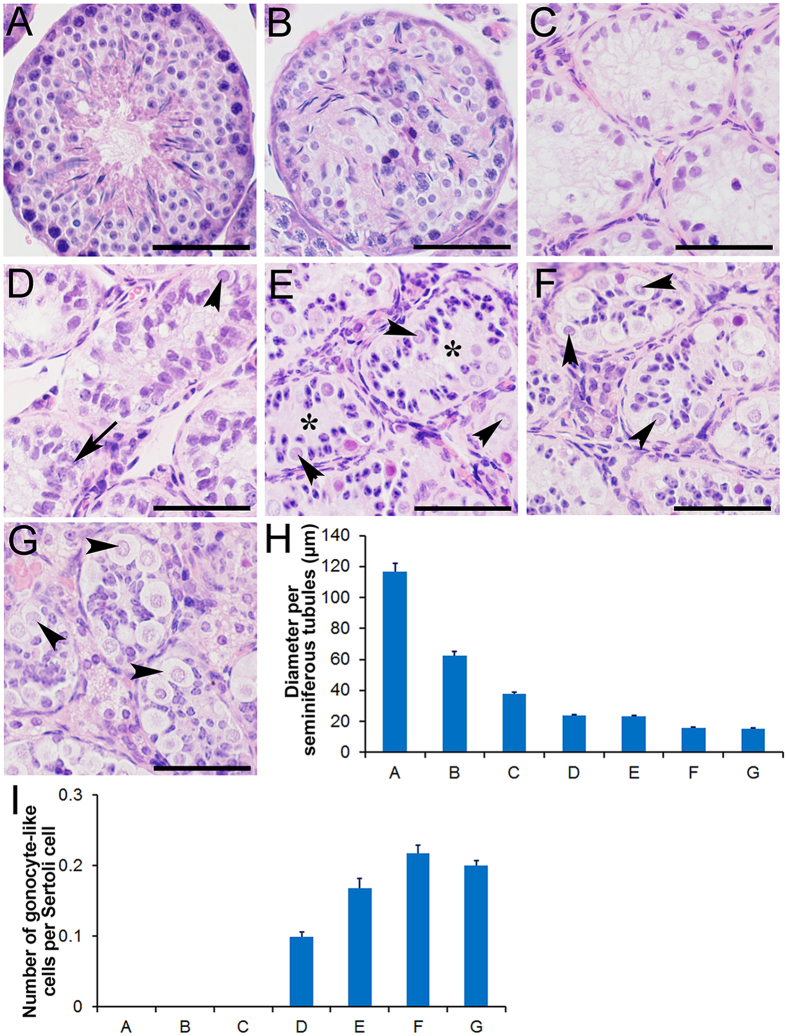
The histological changes in the seminiferous tubules of the plateau pikas between the breeding and non-breeding seasons. The seminiferous tubules of the plateau pikas exhibited a normal spermatogenic epithelium during the breeding season (**A**, defined as ST1). When the testes started to degenerate, the cells in the seminiferous tubules were gradually lost (**B**, defined as ST2) until very few cells were left (**C**, defined as ST3). Large gonocyte-like cells with large round nuclei appeared in the peripheral regions of the seminiferous tubules (**D**, defined as ST4, black arrow heads). Some of the Sertoli cell nuclei showed dark-punctate hematoxylin staining (**D**, black arrow). The seminiferous tubules were composed of large gonocyte-like germ cells (**E**,**F**, defined as ST5 and ST6, black arrow heads) and Sertoli cell nuclei showing dark-punctate hematoxylin staining (**E,F**). Finally, the Sertol cell nuclei migrated to the center of the seminiferous tubules (from **E**,**F**), which resembled the testes of juvenile pikas morphologically (**G**). The stars in (**E**) indicate the centers of the seminiferous tubules that would later be occupied by Sertoli cell nuclei in (**F**). (**H**) summarizes the diameters (mean ± SEM) of the seminiferous tubules shaped as panels (**A–G**). (**I**) summarizes the number of large gonocyte-like cells per Sertoli cells in different types of seminiferous tubules shaped as panels (**A**–**G**). The scale bars represent 30 μm.

To examine histological changes during the transition from the breeding season to the non-breeding season, pika testes were collected at different time points from early June to late July, and their histology was examined with H&E staining. At each time point, 10 adult male pikas were examined. In early June, most of the seminiferous tubules displayed normal histology (Fig. 2A, defined as ST1, approximately 70%). Only a small portion of seminiferous tubules with abnormal histology were observed (Fig. 2B, defined as ST2, approximately 30%). The regression of the seminiferous tubules was evident in mid-June. Only approximately 30% of the seminiferous tubules showed normal histology (ST1), and most of the tubules were undergoing regression (ST2, approximately 30%) or showed severe degeneration (Fig. 2C, defined as ST3, approximately 40%). Severe degeneration of the seminiferous tubules was observed in late June. Approximately 65% of the tubules showed severe degeneration (ST3). As shown in Fig. 2C, spermatids and spermatocytes were completely absent from the seminiferous tubules, and only Sertoli cells and spermatogonia were observed in the peripheral region. Approximately 35% of the tubules were undergoing regression (ST2). Interestingly, a small number of large cells with round nuclei appeared in early July (Fig. 2D, defined as ST4, 26%). The morphology was similar to that of the gonocytes observed in the testes of juvenile pikas (Fig. 2D). The number of gonocyte-like cells per tubule gradually increased, and the number of tubules containing these cells also increased. In mid-July, gonocyte-like cells were observed in approximately 60% of the tubules (Fig. 2E, defined as ST5). By the end of July, approximately 90% of the tubules were as shown in Fig. 2F (defined as ST6). At that time, the number of Sertoli cells per tubule also increased, and the nuclei of the Sertoli cells migrated from the peripheral region to the center of the seminiferous tubules (Fig. 2F), a pattern similar to that observed in the testes of juvenile pikas morphologically (Fig. 2G). These 6 stages (ST1-ST6) were different from the stages of the seminiferous cycle (Supplementary Figure 1). The diameters of the seminiferous tubules, illustrated in Fig. 2A–F, exhibited a continuous decrease from more than 100 μm to less than 20 μm (Fig. 2H). The ratio of gonocyte-like cells to Sertoli cells at each time point was also calculated. As shown in Fig. 2I, the ratio gradually increased from early July (approximately 0.1) to late July (approximately 0.21).

### The number of TUNEL-positive cells did not increase in the degenerated seminiferous tubules during the non-breeding season

To examine cell apoptosis in the pika testes during the non-breeding season, a TUNEL assay was performed. As shown in Supplementary Figure 2, A small number of TUNEL-positive cells were observed in normal spermatogenic tubules (A, ST1). The number of TUNEL-positive cells increased in the degenerating tubules (B, ST2). Additionally, very few TUNEL-positive cells were noted in the seminiferous tubules at ST3 (C) and ST4 (D). In the ST5 (E) and ST6 (F) seminiferous tubules, TUNEL-positive cells were rarely observed.

### AP-2γ was expressed in the gonocyte-like cells of adult pika testes during the non-breeding season

**Figure 3:**
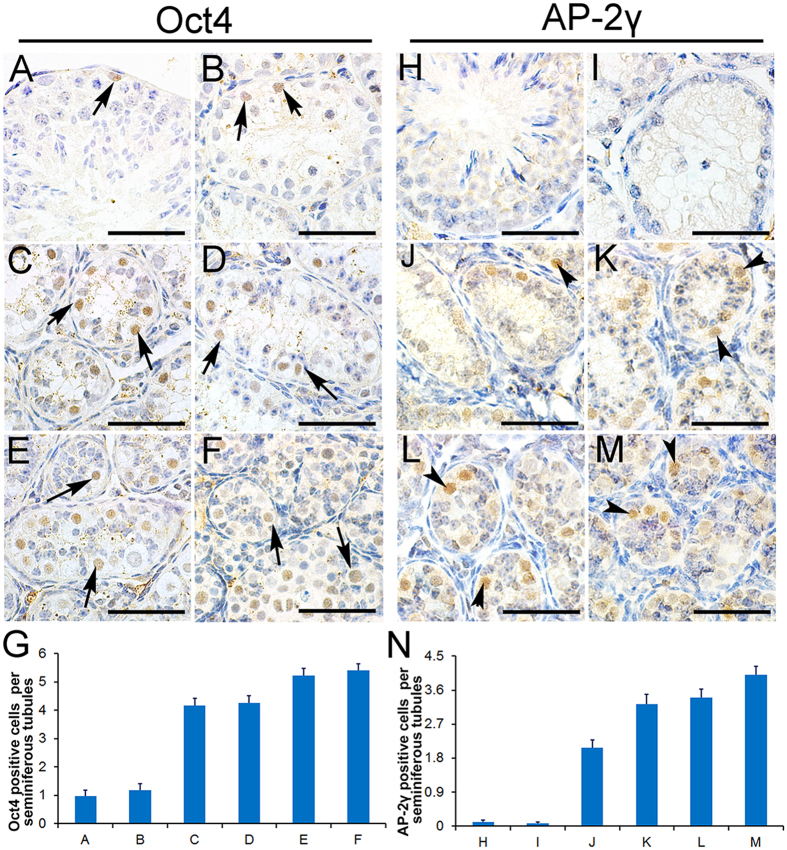
Gonial stem cells express AP-2γ in the testes of non-breeding season pikas. (**A**) Gonial stem cells were labeled using the Oct4 antibody (black arrows) in the testes of the breeding-season pikas. Oct4-positive gonial stem cells were detected in the degenerated pika testes (**B**, black arrows), and the large, gonocyte-like cells with round nuclei were also Oct4 positive in the ST4, ST5, and ST6 pika seminiferous tubules (**C**–**E**, black arrows). (**F**) The gonocytes in the juvenile pika testes were Oct4 positive (black arrows). (**G**) Summary of Oct4-positive cell number (mean ± SEM) per seminiferous tubule (**A–F**). The germ cells from the breeding-season (**H**) and degenerated (**I**) testes were negative for the gonocyte-specific marker AP-2γ. The large, round-nuclei gonocyte-like cells were labeled with AP-2γ in the testes of the ST4, ST5 and ST6 seminiferous tubules in pikas (**J–L**, black arrowheads). The gonocytes in the juvenile pika testes were labeled with the AP-2γ antibody (**M**). Panel (**N**) shows the number of AP-2γ-positive cells (mean ± SEM) per seminiferous tubule, shaped as panels (**H–M**). Scale bars represent 30 μm.

To further identify the gonocyte-like cells in the pika testes, immunohistochemistry was performed using anti-Oct4 and anti-AP-2γ antibodies. Oct4 is a pluripotent marker gene that is expressed in primordial germ cells (PGCs), gonocytes, and undifferentiated spermatogonia. As shown in Fig. 3, the spermatogonia of the normal spermatogenic pikas were labeled with the Oct4 antibody (Fig. 3A). A few Oct4-positive germ cells were also observed in the degenerated seminiferous tubules in late June (Fig. 3B), indicating that some spermatogonia survived in the non-breeding season. The gonocyte-like cells in the ST4-ST6 seminiferous tubules (Fig. 3C–E) and the gonocytes in the testes of juvenile pikas (Fig. 3F) were also labeled with the Oct4 antibody. The number of Oct4-positive cells per tubule was summarized in Fig. 3G.

It has been reported that AP-2γ is a gonocyte-specific marker^17^. AP-2γ protein was detected in the germ cells of juvenile pika testes (Fig. 3M) but not in the germ cells of the normal seminiferous tubules of the adult testes (Fig. 3H) or the degenerated testes in late June (Fig. 3I). Interestingly, the AP-2γ signal was detected in the large gonocyte-like cells observed in the seminiferous tubules from early July to late July (ST4-ST6 tubules, Fig. 3J–L). The number of AP-2γ-positive gonocyte-like cells per tubule gradually increased from late June to late July (Fig. 3N).

### The nuclei of Sertoli cells migrated from the periphery to the center of the seminiferous tubules and began to express AMH during the non-breeding season

**Figure 4:**
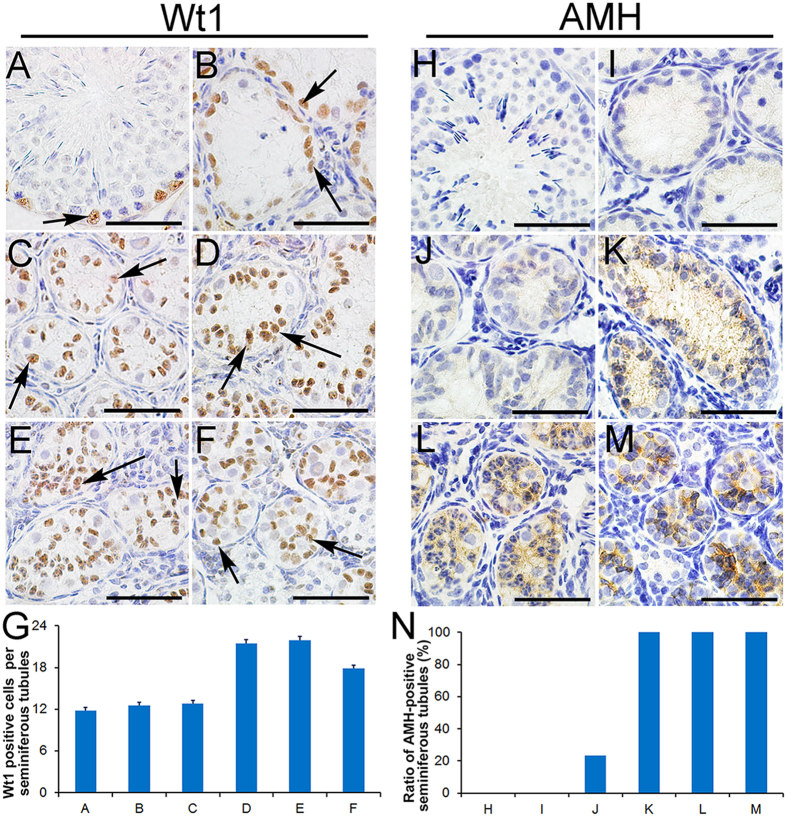
Sertoli cells express AMH in the testes of non-breeding season pikas. The Sertoli cells were labeled with the anti-Wt1 antibody. The nuclei of the Wt1-positive Sertoli cells were localized to the peripheral regions of the seminiferous tubules in the normal spermatogenic (**A**, black arrows) and degenerated (**B**, black arrows) testes of the pikas. The number of Wt1-positive Sertoli cells gradually increased in the ST4, ST5 and ST6 testes, and the nuclei of the Wt1-positive Sertoli cells migrated from the periphery to the center of the seminiferous tubules (**C**–**E**, black arrows), a pattern very similar to that observed in the juvenile pika testes (**F**, black arrows). The Wt1-positive cell number (mean ± SEM) per seminiferous tubules was summarized in (**G**). The expression of AMH was examined via immunohistochemistry. The primitive Sertoli cell marker AMH was absent in the normal spermatogenic (**H**) and the degenerated (**I**) seminiferous tubules of the pikas. The expression of AMH gradually increased in the ST4, ST5, and ST6 testes of the pikas (**J–L**). AMH was highly expressed in the testes of juvenile pikas (**M**). Panel (**N**) shows the ratio of AMH-positive seminiferous tubules shaped as panels (**H**–**M**). Scale bars represent 30 μm.

H&E staining showed that the number of Sertoli cells per tubule increased and that the nuclei of the Sertoli cells migrated from the periphery to the center of the seminiferous tubules in the testes of the non-breeding pikas. To confirm these results, immunostaining was performed using an anti-Wt1 antibody. As shown in Fig. 4, Wt1-positive Sertoli cells were localized at the periphery of the seminiferous tubules in both the normal spermatogenic cells in early June (Fig. 4A) and in the degenerated testes in late June (Fig. 4B). Interestingly, the number of Wt1-positive Sertoli cells per tubule gradually increased from late June to late July, and the nuclei migrated to the centers of the tubules (ST4-ST6, Fig. 4C–E), a pattern similar to that found in the testes of juvenile pikas (Fig. 4F). AMH is expressed in primitive Sertoli cells and is absent in the adult testes^18^. As shown in Fig. 4H–M, AMH protein was detected in the testes of juvenile pikas (Fig. 4M), whereas it was completely absent from the normal spermatogenic cells in early June (Fig. 4H) and degenerated in late June (Fig. 4I) in the seminiferous tubules of the studied pikas. Interestingly, the expression of AMH gradually increased in the Sertoli cells of seminiferous tubules from early July to mid-July (ST4-ST5, Fig. 4J,K), and AMH was abundantly expressed in the Sertoli cells in late July (ST6, Fig. 4L). In the testes of the juvenile pikas, all the seminiferous tubules were AMH-positive (Fig. 4N).

### The de-differentiation-like morphological change of the testes during the non-breeding season is species-dependent

**Figure 5:**
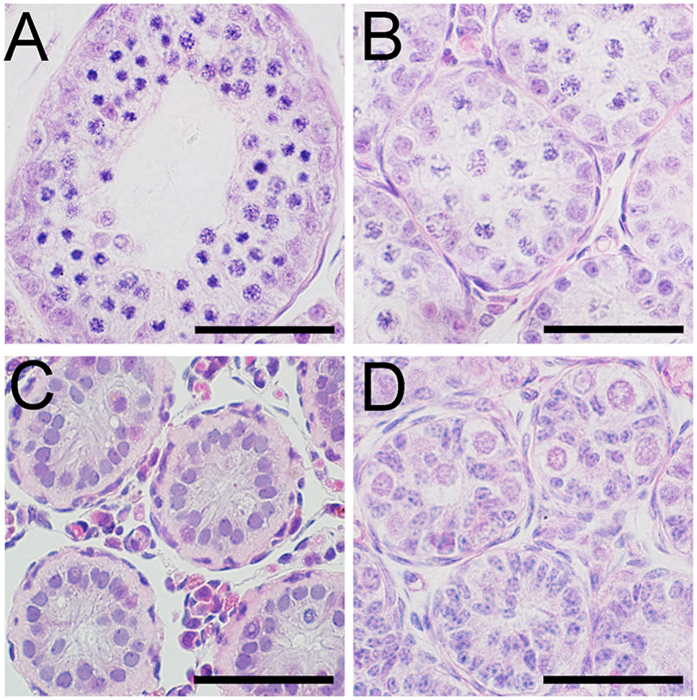
The histology of the testes of various species during the non-breeding season. (**A**) Brandt’s voles *(Lasiopodomys brandtii)*; (**B**) Tolai hares *(Lepus tolai)*; (**C**) Blyth’s voles *(Phaiomys leucurus);* (**D**) Daurian pikas *(Ochotona daurica)*. Scale bars represent 30 μm.

To further explore the potential mechanism leading to the DDL transition of pika testes, the histology of the testes of other seasonally breeding small mammals living in the same environment (Blyth’s voles, Tibetan plateau, altitude = 3970 m) or a different environment (Brandt’s voles, Inner Mongolia, altitude = 1080 m; Tolai hares, Inner Mongolia, altitude = 1037 m; Daurian pikas, Inner Mongolia, altitude = 1242 m) was examined. As shown in Fig. 5, the histology of the testes of the Brandt’s voles was normal during the non-breeding season, and only elongating spermatids were absent (Fig. 5A). In the Tolai hares, both round spermatids and elongated spermatids were lost during the non-breeding season (Fig. 5B). Blyth’s voles live at the same altitude and in the same environment as plateau pikas. In the non-breeding season, most of the differentiated germ cells were lost, and undifferentiated spermatogonia with a nuclear rarefaction zone were retained. However, no large gonocyte-like cells and translocation of the nucleus of Sertoli cells were noted in the seminiferous tubules of the Blyth’s voles during the non-breeding season (Fig. 5C). The testes of Daurian pikas, another type of pika that resides at a low altitude, exhibited a DDL phenomenon that was very similar to what was observed in plateau pikas (Fig. 5D). These results suggest that maintaining the spermatogonia in an undifferentiated stage may be a way to adapt to the high-altitude environment and that the DDL transition of the testes during the non-breeding season is a species-specific phenomenon.

### MAP1-LC3 (LC3) levels dramatically increased in the testes of plateau pikas during the non-breeding season

**Figure 6:**
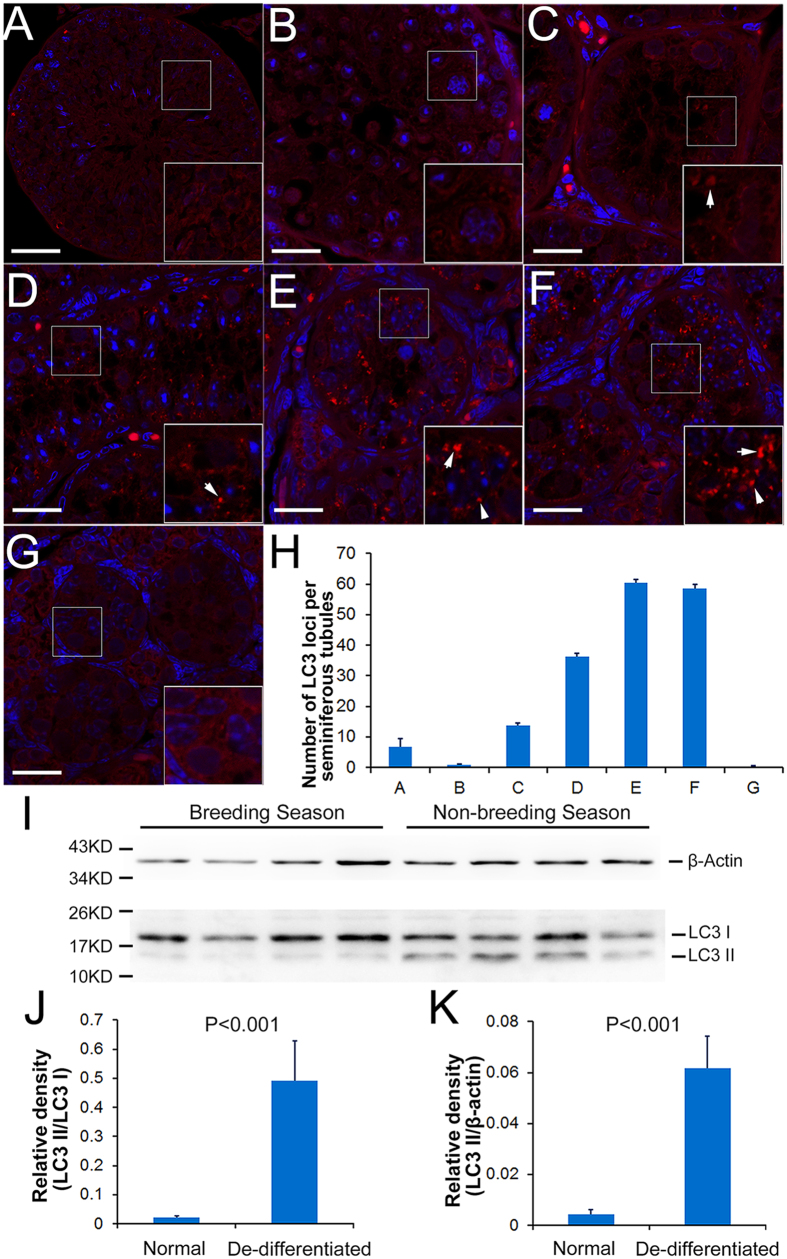
Autophagy levels in normal spermatogenesis and in de-differentiated testes. LC3 immunofluorescence was used to measure autophagy levels in the testes of plateau pikas. LC3 puncta (red) were present but occurred only at a low level in the normal breeding seminiferous tubules (**A**), whereas the puncta level decreased in the degenerating seminiferous tubules (**B**). LC3 puncta recovered in degenerated seminiferous tubules (**C**) and increased in ST4-ST6 tubules (**D–F**). There were almost no LC3 puncta in the testes of juvenile pikas (**G**). Panel (**H**) summarizes the number of LC3 puncta (mean ± SEM) per seminiferous tubule shaped as panels (**A–G**). The bar in panel (**A**) = 40 μm; the bars in panels (**B**–**G**) = 20 μm. Panel (**I**) shows that the protein level of LC3 II markedly increased in the ST5 and ST6 seminiferous tubules. The relative densities of LC3 II/LC3 I and LC3 II/β-actin are illustrated in (**J,K**). The bars in panel (**J,K**) represent ± SEM.

Autophagy is considered to function as a protective mechanism under stressful conditions^19^. To examine whether autophagy is also involved in the de-differentiation of the seminiferous tubules in plateau pikas during the non-breeding season, the expression of LC3 was analyzed via immunofluorescence and Western blotting. As shown in Fig. 6, low levels of LC3 puncta were observed in the normal spermatogenic testes in early June (Fig. 6A), in the degenerating testes in mid-June (Fig. 6B) and in the degenerated testes in late June (Fig. 6C). LC3 puncta dramatically increased in the seminiferous tubules in early July (Fig. 6D), mid-July (Fig. 6E), and late July (Fig. 6F). However, no LC3 puncta were detected in the testes of juvenile pikas (Fig. 6G).

The number of LC3 puncta per tubule at different time points during the non-breeding season was summarized in Fig. 6H. In the seminiferous tubules from early July to late July, the number of LC3 puncta was approximately 5 times higher than that in normal spermatogenic testes. Western blotting analyses further confirmed that LC3 II levels were significantly increased during the non-breeding season compared with the breeding season (Fig. 6I–K). These results suggest that autophagy may be involved in the DDL transition of plateau pika testes during the non-breeding season.

## Discussion

**Figure 7:**
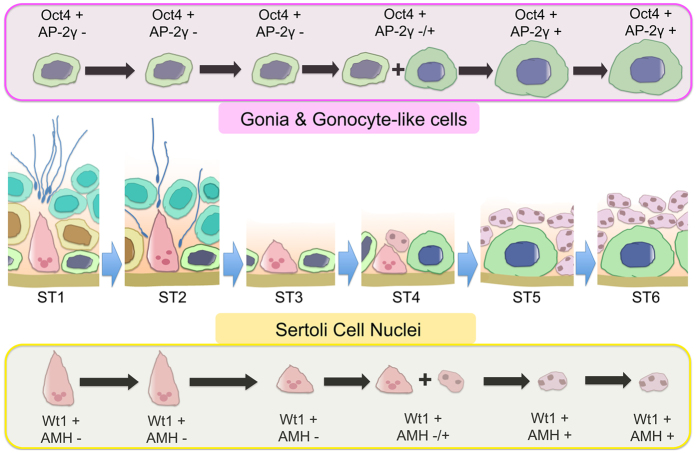
Schematic representation of the spermatogenic epithelium in pikas from the breeding to the non-breeding season. ST1-ST6 represent the 6 stages illustrated in Fig. 2.

For most seasonally breeding wild mammals, the process of spermatogenesis is arrested during the non-breeding season^9^. In this study, we found that the testis of plateau pikas underwent regression during the early non-breeding season, which is consistent with the patterns known in other seasonally breeding mammals^13^,^20^. Histological studies revealed that most of the germ cells were gradually lost, and only a small number of Oct4-positive spermatogonia were observed in the early stage of the non-breeding season. Interestingly, large gonocyte-like germ cells with round nuclei appeared following a massive loss of germ cells in adult pika testes at the end of June. AP-2γ has been reported as a gonocyte-specific marker^17^. In this study, we found that AP-2γ was expressed in the gonocytes of juvenile testes and in large gonocyte-like germ cells in non-breeding adult pika testes but not in breeding adult testes (Fig. 7). These results suggest that gonocytes emerged in the testes of non-breeding plateau pikas. However, the origin of these cells is unclear. In a mouse model, gonocytes are observed only during embryonic stages, and the germ cells start to differentiate within a few days after birth. In contrast, large gonocytes are observed in the testes of juvenile pikas. These results indicate that germ cell development in pikas is different from that in certain rodent models. Therefore, the gonocyte-like germ cells probably de-differentiate from the remaining spermatogonia or directly differentiate from other stem cells existing in the seminiferous tubules; then, most likely, they are developmentally arrested at a certain stage during the non-breeding season. Given that a small number of Oct4-positive spermatogonia remained before gonocyte-like cells were noted, we favor the first possibility.

Sertoli cells are one of the major cell types in testes that provide nutrition and structural support for germ cell development. During embryonic development, primitive Sertoli cells produce anti-Mullerian hormone (AMH), which promotes the degradation of the Mullerian duct^21^,^22^, the female reproductive system precursor. AMH was not expressed in mature Sertoli cells of adult testes, and the nuclei of Sertoli cells were localized at the peripheral regions of the seminiferous tubules. In this study, we found that the nuclei of Sertoli cells moved to the centers of the seminiferous tubules in non-breeding pikas and that AMH was also detected in these cells (Fig. 7), a pattern very similar to that observed in juvenile pika testes. These results indicate that spermatogenesis was arrested and that the morphology and markers in Sertoli cells also changed, a pattern that is also, most likely, a DDL transition during the non-breeding season. However, no direct evidence for this supposition was provided by the present study.

It has been reported that fibroblasts, which are highly differentiated, could be transformed into pluripotent stem cells (iPS) by the overexpression of several transcriptional factors^23^. Other studies have also demonstrated that one cell type could be trans-differentiated into ES-like cells by the overexpression of certain genes^24^,^25^. Spermatogonia could be transformed into ES-like cells under certain culture conditions *in vitro*^26^. These results suggest that these cells have the potential to transform into other cell types by gene reprogramming. Previous studies have also found that cells can undergo *in vivo* de-differentiation during organ regeneration. Examples of this process include the regeneration of the mouse/rat liver after surgical cutting^27^,^28^ and tail/limb regeneration in lizards^29^. In this study, we found a very interesting phenomenon, namely, that the germ cells and Sertoli cells in plateau pika testes during non-breeding season were similar to the cells observed in juvenile pika testes, suggesting possible de-differentiation. This finding provides a new concept on which to base stem cell or/and developmental studies. However, in the present study, we only found that the cell morphology and protein expression were similar to those found in juvenile pikas. More experiments are needed to determine whether this is a real natural de-differentiation process.

One of the most important findings of this study was that the activation of the autophagy pathway was accompanied by de-differentiation of the testes, indicating the potential role of autophagy in this unique process. It has been shown that autophagy is essential for cell differentiation^30^,^31^,^32^. *Atg5*^−/−^ embryos fail to develop to the 4- or 8-cell stage^33^, and *Atg7*^−/−^ hematopoietic stem cells (HSCs) exhibit deficient colony formation^34^,^35^, suggesting that autophagy plays essential roles in cell differentiation. When a cell is undergoing a change in function, autophagy is activated to clear organelles and proteins to adapt to the cell’s new functions. During the non-breeding season, the testes should be in a quiescent state to conserve energy, which requires the clearance of unnecessary cytoplasm and organelles.

In this study, we also found that the DDL transition observed in pikas is a species-specific phenomenon. We found that Daurian pikas, which live in Inner Mongolia, exhibited a DDL transition of the testes similar to that of plateau pikas during the non-breeding season, whereas other seasonally breeding mammals such as Blyth’s voles, which live in the same environment as plateau pikas, and rabbits, which are also lagomorphs, did not exhibit a similar histological transition. Pikas are phylogenetically ancient. In contrast to other small mammals, they neither hibernate nor store food in burrows during the winter. Moreover, to combat the extreme cold weather during the winter (usually below −20 °C), pikas maintain a very high body temperature (approximately 40 °C)^36^, which is unique among all mammals. Due to these constraints, unnecessary organs are highly costly in terms of energy consumption and must be subject to maximal restrictions, and the morphological changes in testes, during which a number of cytoplasm and organelles are cleared, may be a phylogenetically ancient and effective way to reduce energy consumption in winter.

In summary, we found unique morphological changes of both germ cells and Sertoli cells in plateau pikas during the non-breeding season. This phenomenon is most likely a natural de-differentiation process, and autophagy may be involved in this process as a special adaptation of pikas during the non-breeding season. However, further investigations are required to elucidate the detailed mechanisms.

## Methods

### Animals

Plateau pikas were captured in the area southeast of Dawu Town, Maqin County, Qinghai Province, China (at 3908 meters above sea level) using string noose traps^37^. During the breeding and non-breeding seasons, six continuous time points were selected every 10 days in early, mid- and late June and in early, mid- and late July (Fig. 1A). At each time point, 10 adult male pikas were collected. Five additional juvenile pikas were captured during mid-June and mid-July. The age of the pikas was estimated based on their body weight and the state of their fur.

Four male Blyth’s voles *(Phaiomys leucurus*) were captured in late August near Qingzhen Town, Maqin County, Qinghai Province in early August. Four male Brandt’s voles *(Lasiopodomys brandtii)*, four male Daurian pikas *(Ochotona daurica)* and three Tolai hares *(Lepus tolai)* were captured in November near Xilinhaote City, Inner Mongolia. All of these small mammals had been reproductively active during the previous breeding season. This history could be easily determined based on their body weights (the body weight of juvenile animals is much lower). All the experiments were performed in accordance with the guidelines approved by the Ethics Committee of the Northwest Institute of Plateau Biology, Chinese Academy of Sciences, and the Ethics Committee of the Institute of Zoology, Chinese Academy of Sciences.

### Pre-treatment of testis tissue

The captured pikas, rabbits and other rodents were weighed and euthanized quickly in a temporary laboratory near Dawu Town. The pair of testes was collected and weighed separately. The left testis was fixed in Bouin’s fluid, and the right testis was placed in neutral formalin for 24 hours. The fixed testes were dehydrated in 75% ethanol, stored at 4 °C and transported to Beijing on ice.

### Paraffin embedding and H&E staining

In the laboratory in Beijing, the testes fixed in neutral formalin were dehydrated to 100% ethanol via a series concentration of ethanol, cleared in xylene and then embedded in paraffin wax after immersion in the paraffin for 2 h. The testes fixed in Bouin’s fluid were immersed in 75% ethanol for 72 h, with the ethanol refreshed every 12 h to wash out the residual picric acid. The Bouin’s-fluid-fixed testes were then embedded in paraffin using the same method used for the neutral-formalin-fixed testes.

The testes fixed in Bouin’s fluid were stained with hematoxylin and eosin. Briefly, the 5-μm slices were deparaffinized in xylene and rehydrated via a series of concentrations of ethanol to water. The slices were stained with Harris hematoxylin for 5 min, treated briefly with 1% hydrochloric acid dissolved in 75% ethanol and then treated in 0.1% ammonia for approximately 10 s. The sections were then dehydrated in 95% ethanol, counterstained by 0.1% eosin for 20 s and mounted in neutral balsam.

### Immunohistochemistry

The testes were sectioned into 5-μm slices and then deparaffinized, rehydrated in water through a series of concentrations of ethanol, and retrieved in 0.01 M citrate buffer (pH = 6.0; for Oct4 and Wt1 staining) or 0.001 M EDTA buffer (pH = 9.0; for AP-2γ and AMH staining) for 15 min. The sections were subsequently immersed in 1% H_2_O_2_ for 10 min and incubated with the primary antibody (in 1% bovine serum albumin) at 4 °C overnight. After washing in PBS, the sections were incubated with HRP-conjugated secondary antibodies for 30 min at room temperature. DAB was used as the substrate for final visualization. The slides were counterstained with Harris hematoxylin. The antibodies used in this study were as follows: goat polyclonal antibody against AMH (SC-6886, Santa Cruz Biotechnology Inc., Santa Cruz, California, USA), rabbit polyclonal antibody against AP-2γ (SC-8977, Santa Cruz Biotechnology Inc., Santa Cruz, California, USA), rabbit monoclonal antibody against Oct4 (#2840S, Cell Signaling Technology, Boston, Massachusetts, USA) and rabbit monoclonal antibody against Wt1 (#2797-1, Epitomics, Burlingame, California, USA).

### TdT-mediated dUTP-biotin nick-end labeling (TUNEL) assay

Cell apoptosis in seminiferous tubules was analyzed with an *in situ* Cell Death Detection Kit (11684809910, Roche, Basel, Switzerland) according to the manufacturer’s instructions. DAB was used as the substrate for color development. Slides were counterstained as described in the immunohistochemistry section of the Methods.

### Immunofluorescence

The paraffin wax-embedded sections were deparaffinized, rehydrated in water through a series of concentrations of ethanol and retrieved in 0.01 M citrate buffer for 15 min to be treated with the primary antibodies. The frozen sections were dried at 55 °C for 5 min and subsequently fixed in 4% PFA for 30 min and treated with 10% bovine serum albumin (BSA) for 1 hour for the primary antibody treatment. The dewaxed and frozen sections were then incubated with a rabbit monoclonal antibody against LC3 (ab58610, Abcam, Shanghai, China) in 1% BSA at 4 °C overnight. After washing, the sections were incubated with TRITC-conjugated secondary antibodies for 30 min at room temperature. DAPI staining to visualize the nucleus was conducted for 10 min. The slides were mounted using glycerol and observed under an LSM 780 confocal microscope (Zeiss, Germany).

### Western blotting

The testis tissues were homogenized in 1 mL of RIPA lysis buffer (pH = 7.4) and incubated for 30 min on ice. After incubation, the homogenate was centrifuged at 12,000 × *g* for 15 min, and the supernatant was collected for further analyses. Approximately 20 μg of the protein extract was loaded per lane in a 15% gel. The gels were run at a constant voltage of 120 V and transferred at a constant current of 240 mA for 90 min. After transfer, the membranes were washed with PBST and blocked in 5% skim milk for 1 h. The LC3 antibody (L7543, Sigma, Shanghai, China) was diluted in PBST (1:1,000) and incubated with the membrane at 4 °C overnight. The blots were finally detected using an Enhanced Chemiluminescence System (ECL) according to the manufacturer’s instructions. The grayscale density was scanned using Image-Pro (version 6.5) software. A Mann-Whitney *U*-test was performed to detect the differences in grayscale values between breeding and non-breeding seasons (SPSS17.0).

## Additional Information

**How to cite this article**: Liu, M. *et al*. Changes in the morphology and protein expression of germ cells and Sertoli cells in plateau pikas testes during non-breeding season. *Sci. Rep.* **6**, 22697; doi: 10.1038/srep22697 (2016).

## Supplementary Material

Supplementary Information
