# Supplementary material for: Changes in the morphology and protein expression of germ cells and Sertoli cells in plateau pikas testes during non-breeding season
Source: Sci Rep. 2016 Mar 4;6:22697. doi: 10.1038/srep22697 (PMC4778026; doi:10.1038/srep22697)

Changes in the morphology and protein expression of germ cells and Sertoli cells in plateau pikas testes during non-breeding season

Ming Liu[1,a], Guangming Cao[1,4,a], Yanming Zhang[3], Jiapeng Qu[3], Wei Li[1], Xinrong Wan[2], Yu-xia Li[1], Zhibin Zhang[2,*], Yan-ling Wang[1,*], and Fei Gao[1,*]

1 State Key Laboratory of Stem Cells and Reproductive Biology, Institute of Zoology, Chinese Academy of Sciences, Beijing 100101, P.R. China

2 State Key Laboratory of Integrated Management of Pest Insects and Rodents, Institute of Zoology, Chinese Academy of Sciences, Beijing 100101, P.R. China

3 Key Laboratory of Qinghai-Tibetan Plateau Biological Adaptation and Evolution, Northwest Institute of Plateau Biology, Chinese Academy of Sciences, Xining, 810008, Qinghai Province, P.R. China

4 University of the Chinese Academy of Sciences, Beijing 100049, P.R. China

a. Equal contribution

* Corresponding authors: Fei Gao (gaof@ioz.ac.cn), Zhibin Zhang (zhangzb@ioz.ac.cn) and Yan-ling Wang (wangyl@ioz.ac.cn).

Supplementary Figures

Supplementary Figure 1. The primary description of the seminiferous cycle in plateau pikas.

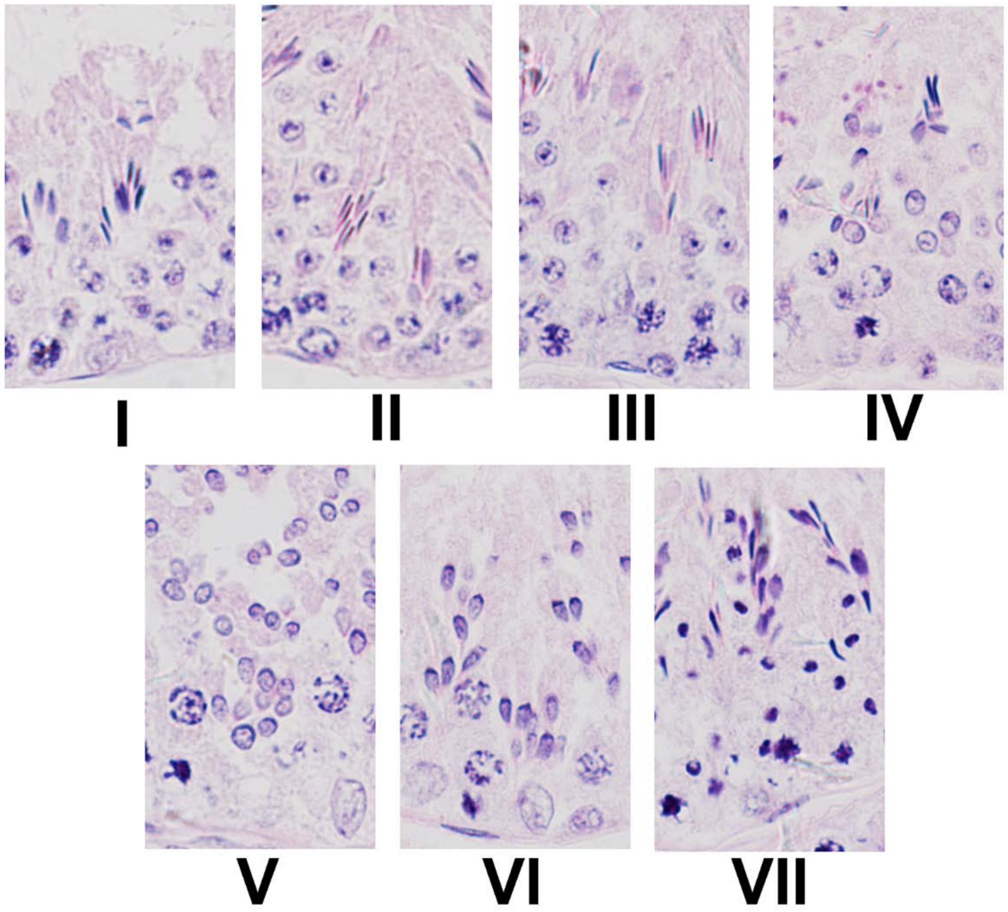

Supplementary Figure 2. TUNEL-positive staining mainly located in the spermatocytes and spermatids in the degenerated seminiferous tubules. (A) normal spermatogenic (ST1) tubule; (B) degenerating (ST2) tubule; (C) degenerated (ST3) tubule; (D-F) DDL (ST4-ST6) tubules. Arrows indicate TUNEL-positive signaling. The bars represent 30 μm.

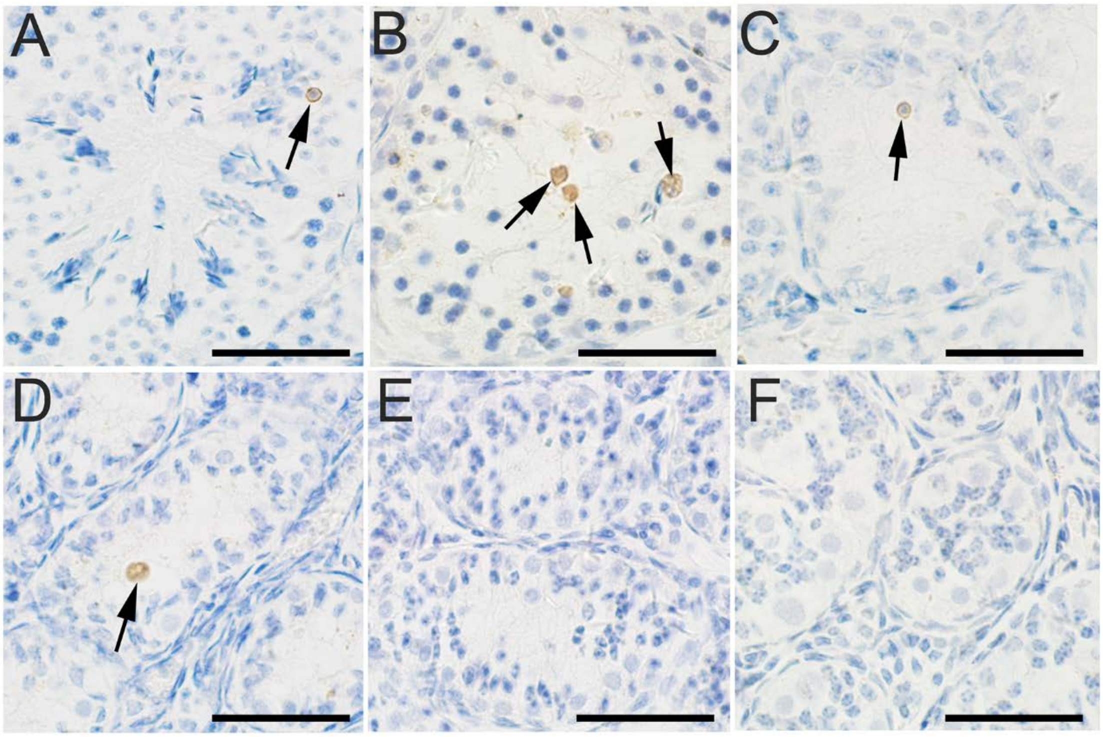

Supplement: Supplementary Information [file srep22697-s1.pdf]
